# Shortcuts to adiabatic passage for fast generation of Greenberger-Horne-Zeilinger states by transitionless quantum driving

**DOI:** 10.1038/srep15616

**Published:** 2015-10-28

**Authors:** Ye-Hong Chen, Yan Xia, Jie Song, Qing-Qin Chen

**Affiliations:** 1 Department of Physics, Fuzhou University, Fuzhou 350002, China; 2 Department of Physics, Harbin Institute of Technology, Harbin 150001, China; 3 Zhicheng College, Fuzhou University, Fuzhou 350002, China

## Abstract

Berry’s approach on “transitionless quantum driving” shows how to set a Hamiltonian which drives the dynamics of a system along instantaneous eigenstates of a reference Hamiltonian to reproduce the same final result of an adiabatic process in a shorter time. In this paper, motivated by transitionless quantum driving, we construct shortcuts to adiabatic passage in a three-atom system to create the Greenberger-Horne-Zeilinger states with the help of quantum Zeno dynamics and of non-resonant lasers. The influence of various decoherence processes is discussed by numerical simulation and the result proves that the scheme is fast and robust against decoherence and operational imperfection.

“Shortcuts to adiabatic passage (STAP)”^1^,^2^ which are a set of techniques to speed up a slow quantum adiabatic process usually through a non-adiabatic route, have attracted a great deal of attention in recent years. They can overcome the harmful effects caused by decoherence, noise or losses because of a long operation time. Quantum science also greatly desires fast and robust theoretical methods since high repetition rates contribute to the achievement of better signal-to-noise ratios and better accuracy. Therefore, in the last several years, STAP have been applied in a wide range of systems in theory and experiment^3^,^4^,^5^,^6^,^7^,^8^,^9^,^10^,^11^,^12^,^13^,^14^,^15^,^16^,^17^,^18^,^19^,^20^,^21^,^22^,^23^. Various reliable, fast and robust methods and schemes have been proposed to implement quantum information processing (QIP), such as fast population transfer^5^,^6^,^7^, fast entanglement generation^6^,^8^, fast implementation of quantum phase gates^9^.

To construct shortcuts to speed up adiabatic processes effectively, two methods which are in fact strongly related, and even potentially equivalent to each other^24^: are invariant-based inverse engineering based on Lewis-Riesenfeld invariant^10^,^25^ and Berry’s approach named “transitionless quantum driving” (TQD)^26^,^27^,^28^,^29^. Whereas, each of the two methods also has its own characteristics, for example, using Lewis-Riesenfeld invariants to construct shortcuts usually does not have to break down the form of the original Hamiltonian *H*_0_(*t*), so that the possibility of designing a Hamiltonian *H*(*t*) very difficult or impossible to implement in practice is avoided^5^,^12^. However, the invariants always have fixed forms which lead to that shortcut methods based on Lewis-Riesenfeld invariants might be limited or even hopeless in some cases to construct shortcuts to implement QIP rapidly^5^. For example, in the paper^8^ proposed by Chen *et al*., they had no choice but to make one of the atoms to be a control qubit or use auxiliary levels for the atoms to generate entangled states.

There is still plenty to do to make wide applications of STAP for fast QIP in some experimental systems, for example, the cavity quantum electronic dynamics (QED) systems. It is worth noting that, TQD provides a very effective method to construct the “counter-diabatic driving” (CDD) Hamiltonian *H*(*t*) which accurately drives the instantaneous eigenstatees of *H*_0_(*t*). Nevertheless, it is almost always found that the designed CDD Hamiltonian is hard to be directly implemented in practice^2^,^30^,^31^,^32^,^33^,^34^, especially in multiparticle systems. Examples of ways to overcome this problem may be found in refs 34, 35, 36, 37, 38. Also, in a large detuning limit, Lu *et al*.^6^ have found a simplified effective Hamiltonian equivalent to *H*(*t*). This idea inspires us that finding an alternative physically feasible (APF) Hamiltonian which is effectively equivalent to *H*(*t*). However, the approximation in ref. 6 is too complex to be generalized to *N*-qubit entanglement cases. It is known to all that, entanglement of more qubits shows more nonclassical effects and is more useful for quantum applications. For example, one of the two kinds of three-qubit entangled states named the Greenberger-Horne-Zeilinger (GHZ) states provide a possibility for testing quantum mechanics against local hidden theory without using Bell’s inequality^39^,^40^. Therefore, great interest has arisen regarding the significant role of the GHZ states in the foundations of quantum mechanics measurement theory and quantum communication. In view of that we wonder if it is possible to use TQD to construct shortcuts for one-step generation of multi-qubit entanglement, i.e., the three-atom GHZ states, without abandoning any of the atoms or using auxiliary levels.

In this scenario, motivated by refs 5, 6, 7, 8, we use TQD to construct STAP to generate the three-atom GHZ states effectively and rapidly in one step. It would be a promising idea of applying STAP to realize multi-qubit entanglement generation in cavity QED systems. Different from ref. 6, we use the quantum Zeno dynamics^41^,^42^ to simplify the system first and then under the large detuning conditon, we obtain the effective Hamiltonian which is equivalent to the corresponding CDD Hamiltonian to speed up the evolution process. Therefore, the adiabatic process for a multi-qubit system is speeded up, and the STAP is easy to be achieved in experiment. Comparing with ref. 8, we use TQD in this paper so that the laser pulses are not strongly limited and we do not need to use auxiliary levels or multi-step operations to generate the three-atom GHZ states. Moreover, we find that any quantum system whose Hamiltonian is possible to be simplified into the form in eq. (15), the corresponding APF Hamiltonian can be built and the STAP can be constructed with the same approach presented in this paper. The above advantages mean the present scheme is much more useful in dealing with the fast and noise-resistant generation of multi-qubit entanglement or even other QIP.

## Basic theories

### Transitionless quantum driving

Consider an arbitrary time-dependent Hamiltonian *H*_0_(*t*), with instantaneous eigenstates and energies given by


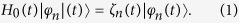


When this system satisfies the adiabatic condition, *H*_0_(*t*) will drive the system into


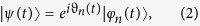


where


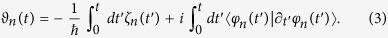


To find the Hamiltonian *H*(*t*) that drives the eigenstates 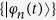, we define a unitary operator


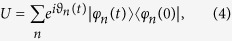


which obeys


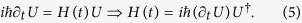


Then the Hamiltonian *H*(*t*) is obtained


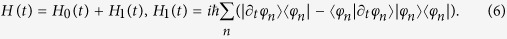


The simplest choice is 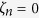, for which the bare states 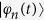, with no phase factors, are driven by^26^


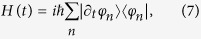


reflecting


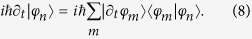


### Quantum Zeno dynamics

The quantum Zeno dynamics was named by Facchi and Pascazio in 2002^42^. It is derived from the quantum Zeno effect which describes a phenomenon that the system can actually evolve away from its initial state while it still remains in the so-called Zeno subspace determined by the measurement when frequently projected onto a multidimensional subspace. According to von Neumann’s projection postulate, the quantum Zeno dynamics can be achieved via continuous coupling between the system and an external system instead of discontinuous measurements^42^. In general, we assume that a dynamical evolution process is governed by the Hamiltonian


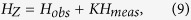


where 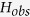 is the Hamiltonian of the quantum system investigated, *K* is a coupling constant, and 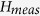 is viewed as an additional interaction Hamiltonian performing the measurement. In the “infinitely strong measurement” limit 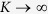
41,42, The Hamiltonian for the whole system is nearly equivalent to


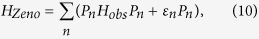


whit *P*_*n*_ being the *n*th orthogonal projection onto the invariant Zeno subspace 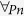 and the eigenspace of 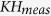 belonging to the eigenvalue *ε*_*n*_, i.e., 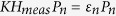.

## Model

**Figure 1:**
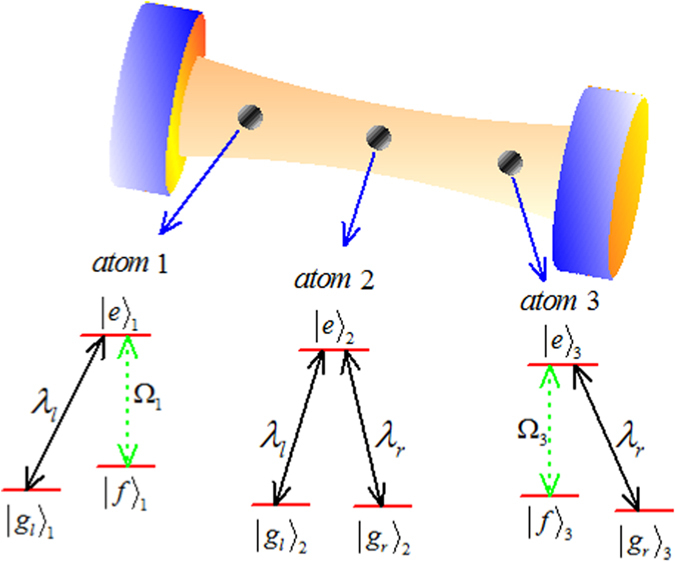
The cavity-atom combined system and the atomic level configuration for the original Hamiltonian.

We consider three Λ-type atoms are trapped in a bimodal-mode cavity as shown in Fig. 1. Atoms 1, 2, and 3 have three sets of ground states 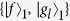, 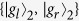, and 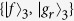, respectively, and each of them has an excited state 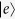. The atomic transition 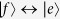 is driven resonantly through classical laser field with time-dependent Rabi frequency Ω(*t*), transition 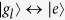 is coupled resonantly to the left-circularly polarized mode of the cavity with coupling λ_*l*_, and transition 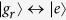 is coupled resonantly to the right-circularly polarized mode of the cavity with coupling λ_*r*_. Under the rotating-wave approximation (RWA), the interaction Hamiltonian for this system reads 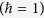:


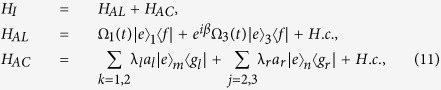


where *H*_*AL*_ denotes the coupling between the atoms and the laser pulses, and *H*_*AC*_ denotes the coupling between the atoms and the cavities, *a*_*l*_ and *a*_*r*_ are the left- and right-circularly annihilation operators of the cavity modes, and *β* means the two Rabi frequencies are *β*-dephased from each other. If we assume the initial state is 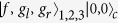, the system will evolve within a single-excitation subspace with basis states


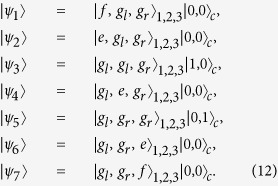


In light of quantum Zeno dynamics, we rewrite the Hamiltonian *H*_*I*_ in eq. (11) as *H*_*re*_ through the relation 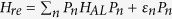 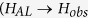 and 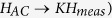, where


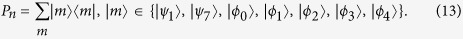


Here 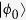, 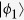, 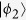, 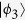, and 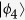 are the eigenvectors of *H*_*AC*_ corresponding eigenvalues 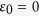, 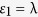, 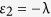, 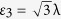, and 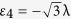, respectively. And we obtain (we set 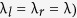



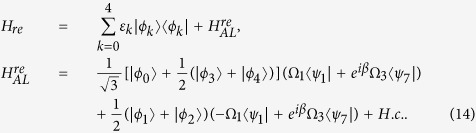


Through performing the unitary transformation 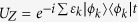 and neglecting the terms with high oscillating frequency by setting the condition 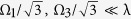 (the Zeno condition), we obtain an effective Hamiltonian


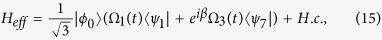


which can be seen as a simple three-level system with an excited state 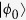 and two ground states 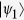 and 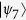. For this effective Hamiltonian, its eigenstates are easily obtained


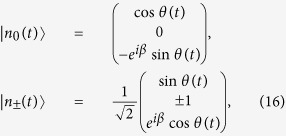


corresponding eigenvalues 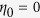, 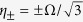, respectively, where 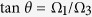 and 
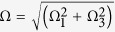
. When the adiabatic condition 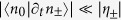 is fulfilled, the initial state 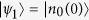 will follow 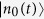 closely, and when 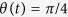 and 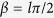 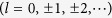, we obtain the GHZ states: 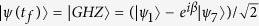. When *β* = *π*, it shows the most common form: 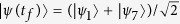. However, this process will take quite a long time to obtain the target state, which is undesirable.

## Using TQD to construct shortcuts to adiabatic passage

The instantaneous eigenstates 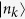 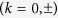 for the effective Hamiltonian 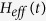 above do not satisfy the Schrödinger equation 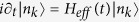. According to Berry’s general transitionless tracking algorithm^26^, from 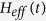, one can reverse engineer *H*(*t*) which is related to the original Hamiltonian 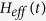 but drives the eigenstates exactly. From refs 6,11,12, we learn the simplest Hamiltonian *H*(*t*) is derived in the form


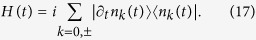


**Figure 2:**
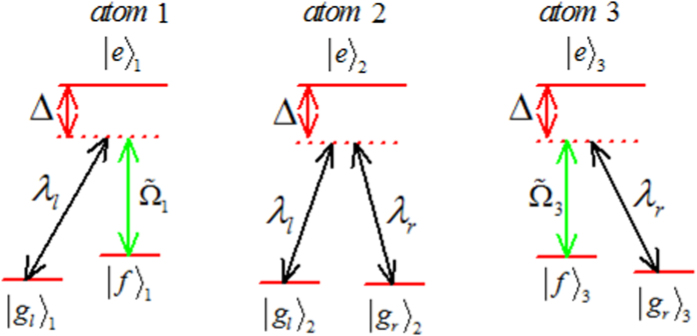
The atomic level configuration for the APF Hamiltonian.

Substituting eq. (16) in eq. (17), we obtain


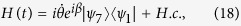


where 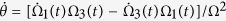. Similar to ref. 6, for this three-atom system in a real experiment, the Hamiltonian *H*(*t*) is hard or even impossible to be implemented in practice. We should find an APF Hamiltonian whose effect is equivalent to *H*(*t*). The model used for the APF Hamiltonian is similar to that in Fig. 1 with three atoms trapped in a cavity, and the atomic level configuration is shown in Fig. 2: the transition 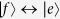 is non-resonantly driven by classical field with time-dependent Rabi frequency 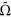 and detuning Δ, the transition 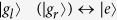 is coupled non-resonantly to the cavity with coupling λ_*l*_ (λ_*r*_) and detuning Δ. The rotating-frame Hamiltonian reads


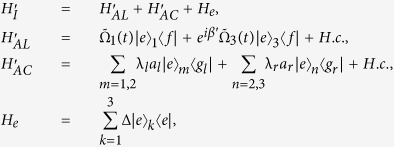


where 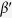 is the phase difference between 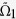 and 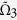. Then similar to the approximation for the Hamiltonian from eq. (11) to eq. (15), we also obtain an effective Hamiltonian for the present non-resonant system^43^


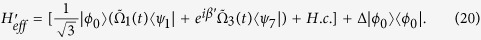


By adiabatically eliminating the state 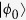 under the condition 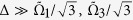, we obtain the final effective Hamiltonian


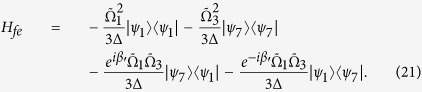


Choosing 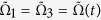, the first two terms of eq. (21) can be removed, and the Hamiltonian becomes


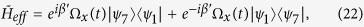


where 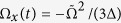. This effective Hamiltonian is equivalent to the CDD Hamiltonian *H*(*t*) in eq. (18) when


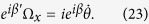


Hence, the Rabi frequencies for the APF Hamiltonian are designed


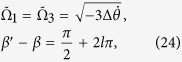


where 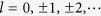.

## Fast and noise-resistant generation of the three-atom GHZ states with STAP

**Figure 3:**
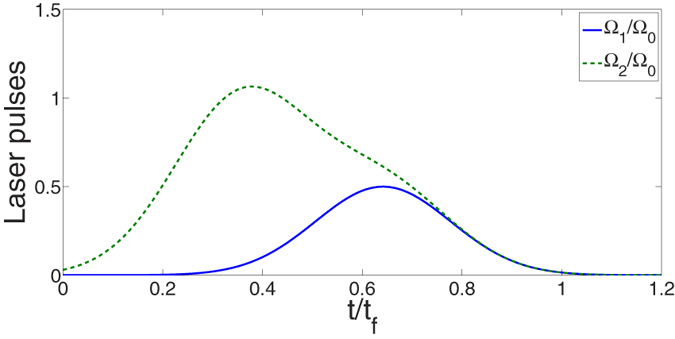
Dependence on *t*/*t*_*f*_ of Ω_1_/Ω_0_ and Ω_3_/Ω_0_.

**Figure 4:**
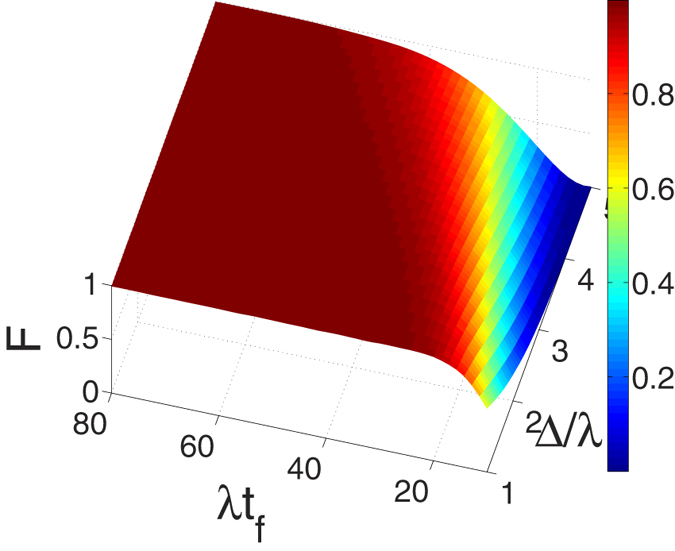
The fidelity *F* of the target state 
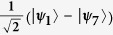
 versus the interaction time λ*t*_*f*_ and the detuning Δ/λ.

**Figure 5:**
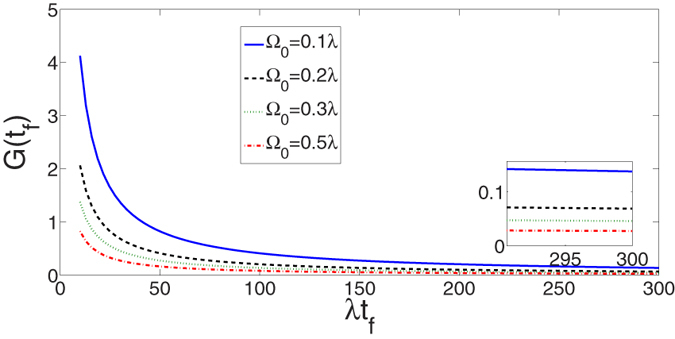
Time evolution of the populations for the states 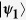 and 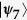 with 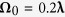, *t*_*f*_ = 35/λ and Δ  = 2.2λ, (a) governed by the APF Hamiltonian 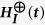, (b) governed by the original Hamiltonian *H*_*I*_(*t*).

**Figure 6:**
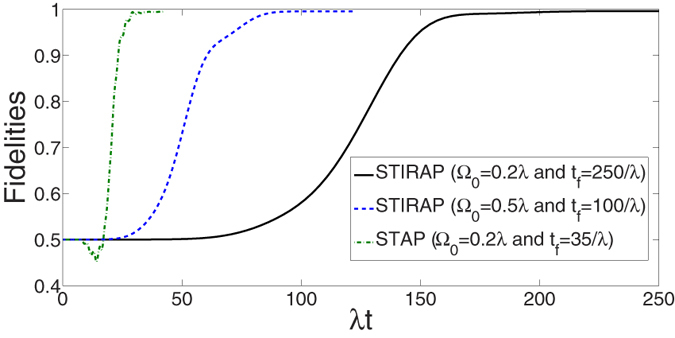
The relationship between *G*(*t*_*f*_) and λ*t*_*f*_ for testing the adiabatic condition.

**Figure 7:**
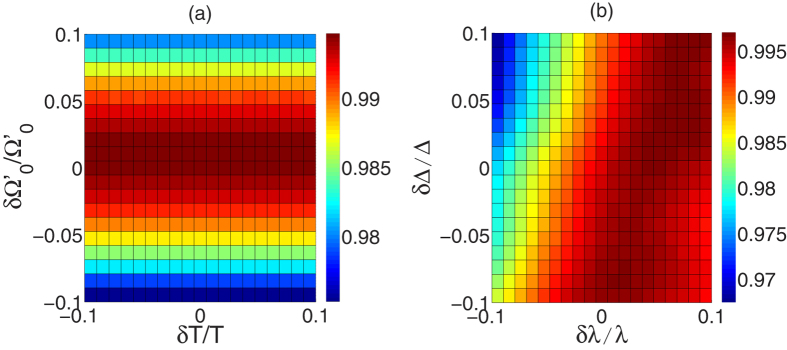
The comparison between the fidelities of the three-atom GHZ state governed by the APF Hamiltonian 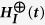 and the original Hamiltonian *H*_*I*_(*t*).

We will show that the creation of a three-atom GHZ state governed by 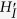 is much faster than that governed by *H*_*I*_. To satisfy the boundary condition of the fractional stimulated Raman adiabatic passage (STIRAP),


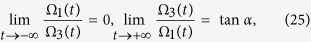


the Rabi frequencies 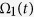 and 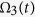 in the original Hamiltonian *H*_*I*_(*t*) are chosen as


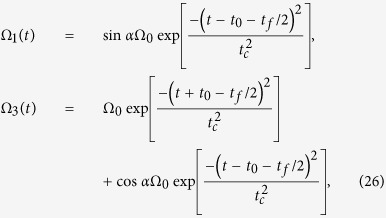


where Ω_0_ is the pulse amplitude, *t*_*f*_ is the operation time, and *t*_0_, *t*_*c*_ are some related parameters. In order to create a three-atom GHZ state, the finial state 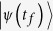 should be 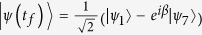 according to eq. (16). Therefore, we have tan*α* = 1. By choosing parameters for the laser pulses suitably to fulfill the boundary condition in eq. (25), the time-dependent 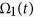 and 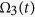 are gotten as shown in Fig. 3 with parameters 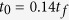 and 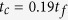. For simplicity, we set *β* = 0 in the following discussion. Fig. 4 shows the relationship between the fidelity of the generated three-atom GHZ state (governed by the APF Hamiltonian 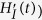 and two parameters Δ and *t*_*f*_ when 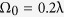 satisfying the Zeno condition, where the fidelity for the three-atom GHZ state is given through 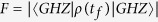 (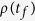 is the density operator of the whole system when 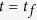). We find that there is a wide range of selectable values for parameters Δ and *t*_*f*_ to get a high fidelity of the three-atom GHZ state. The fidelity increases with the increasing of *t*_*f*_ while decreases with the increasing of Δ. It is not hard to understand, putting eq. (26) into eq. (24) and setting 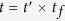, we can find


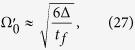


where 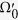 is the amplitude of 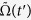. That means, in order to satisfy the Zeno condition 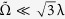 and the large detuning condition 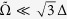, the ratio Δ/*t*_*f*_ should be small enough. Moreover, this relationship also explains the phenomenon in Fig. 4 that to achieve a high fidelity with a larger detuning Δ, a longer interaction time *t*_*f*_ is required. Then to prove the operation time required for the creation of the three-atom GHZ state governed by 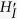 is much shorter than that governed by *H*_*I*_, we contrast the performances of population transfer from the initial state 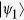 governed by the APF Hamiltonian 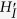 and that governed by the original Hamiltonian *H*_*I*_ in Fig. 5 with 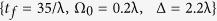. The time-dependent population for any state 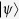 is given by the relationship 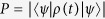, where *ρ*(*t*) is the corresponding time-dependent density operator. The comparison of Fig. 5(a,b) shows that with this set of parameters, the APF Hamiltonian 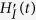 can govern the evolution to achieve a near-perfect three-atom GHZ state from state 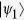 in short interaction time while the original Hamiltonian 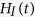 can not. In fact, through solving the adiabatic condition 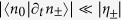, we obtain


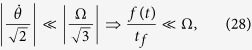


where *f*(*t*) is a wave function whose amplitude is irrelevant to *t*_*f*_. The result shows when Ω_0_ is a constant, the longer the operation time *t*_*f*_ is, the better the adiabatic condition is satisfied. This is proved in Fig. 6. Figure 6 reveals the relationship between *G*(*t*_*f*_) and λ*t*_*f*_, where 
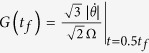
. From this figure, we discover that even with 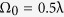 which does not meet the Zeno condition, the operation time required for the three-atom GHZ state generation in an adiabatic system is longer than 100/λ (when 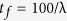, 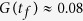). We also plot the fidelities of the evolved states governed by 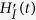 and 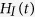 (in different cases) in Fig. 7, with respect to the target three-atom GHZ state. Shown in the figure, even with a large laser intensity, say, 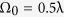, the interaction time required for creation of the three-atom GHZ state via adiabatic passage is still much longer than that via STAP. Generally speaking, the adiabatic condition is satisfied much better with a relatively larger laser intensity, while, the system would be very sensitive to the decoherence caused by the cavity decay with a relatively large laser intensity. This will be proved in the following.

**Figure 8:**
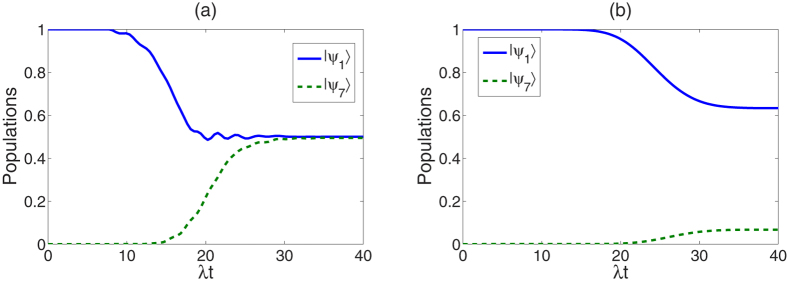
(**a**) Dependences on *κ*/λ and *γ*/λ of the fidelity of the three-atom GHZ state governed by the APF Hamiltonian 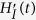 when 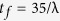, 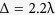 and 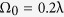. (**b**) Dependences on *κ*/λ and 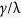 of the fidelity of the three-atom GHZ state governed by the original Hamiltonian *H*_*I*_(*t*) when 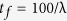 and 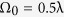.

Once the dissipation is considered, the evolution of the system can be modeled by a master equation in Lindblad form,


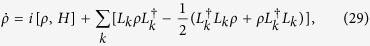


where *L*_*k*_’s are the Lindblad operators. For both the resonant and non-resonant systems, there are eight Lindblad operators governing the dissipation:


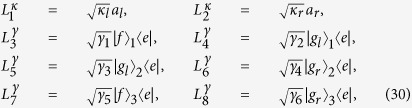


where 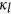 and 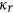 are the decays of the cavity modes, and *γ*_*n*_ 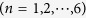 are the spontaneous emissions of atoms. For simplicity, we assume 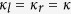, and 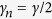. Figure 8(a) shows the fidelity of the three-atom GHZ state governed by the APF Hamiltonian 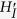 versus these two noise resources with 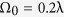, 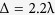, and 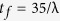. It turns out that the present shortcut scheme with this set of parameters is much more sensitive to the cavity decays than the spontaneous emissions. Ref. 5 contributes to understanding this phenomenon, in fact, with this set of parameters, the Zeno condition for the non-resonant system is not ideally fulfilled because shortening the time implies an energy cost^12^,^24^ (in this system, the energy cost denotes requiring relative-large laser intensities). Known from ref. 5, destroying the Zeno condition slightly is also helpful to achieve the target state in a much shorter interaction time. However, if the Zeno condition has not been satisfied very well, the intermediate states including the cavity-excited states would be populated during the evolution, which causes that the system is sensitive to the cavity decays. However, we can find in Fig. 8(b) which shows fidelity of the three-atom GHZ state governed by original Hamiltonian *H*_*I*_ with 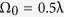 and 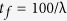 in the presence of decoherence, with large laser intensities, the adiabatic scheme is also sensitive to the cavity decays as we mentioned above. The comparison of these two figures drops a result that the present shortcut scheme is almost the same with the adiabatic one in restraining the decoherence.

**Figure 9:**
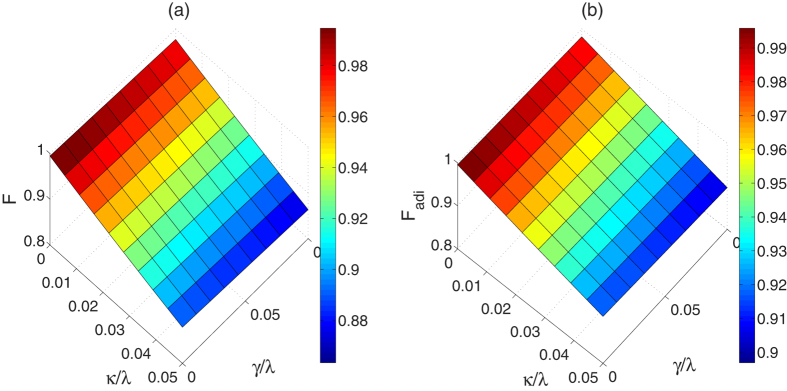
The fidelity *F* of the GHZ state versus the variations of (a) *T* and 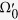. (**b**) λ and Δ.

The robustness against operational imperfection is also a main factor for the feasibility of the scheme because most of the parameters are hard to accurately achieve in experiment. Therefore, we define 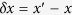 as the deviation of any parameter *x*, where 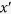 is the actual value and *x* is the ideal value. Then in Fig. 9(a) we plot the fidelity of the GHZ state versus the variations in total operation time *T* 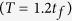 and laser amplitude 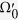, and in Fig 9(b) we plot the fidelity of the GHZ state versus the variations in coupling λ and detuning Δ. As shown in the figures, the scheme is robust against all of these variations. Any deviation 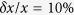 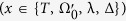 causes a reduction less than 3% in the fidelity.

In a real experiment, the cesium atoms which have been cooled and trapped in a small optical cavity in the strong-coupling regime^44^,^45^ can be used in this scheme. We take the hyperfine states of 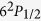 as the excited sates and the hyperfine states of 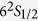 as the ground sates. With a set of cavity QED parameters 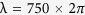 MHz, 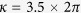 MHz, and 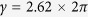 MHz in strong-coupling regime^46^,^47^,^48^, the fidelity of the three-atom GHZ state in this paper is 98.24%. Thus, the scheme is robust and might be promising within the limits of current technology.

## Conclusion

We have presented a promising method to construct shortcuts to adiabatic passage (STAP) for a three-atom system to generate GHZ states in the cavity QED system. Through using quantum Zeno dynamics and “transitionless quantum driving”, we are free to simplify a complicated Hamiltonian and choose the laser pulses to construct shortcuts in multi-qubit system to implement the fast quantum information processing. Numerical simulation demonstrates that the scheme is fast and robust against the decoherence caused by both atomic spontaneous emission, photon leakage and operational imperfection. The deficiency is that the present scheme might be sensitive to the cavity decays because of some inevitable factors. Compared with the previous shortcut methods, this method obviously works better at entanglement generation in multi-qubit systems. In fact, any quantum system whose Hamiltonian is possible to be simplified into the form in eq. (15), the shortcut can be constructed with the same method presented in this paper. For example, similar to refs 49,50 for the generation of the multiparticle GHZ states in an atom-fiber-cavity combined system, we can shorten the operation time using the same method in the following steps: (1) We consider the Hamiltonian *H*_*AL*_ (the Hamiltonian describing the interaction between atoms and lasers) as *H*_*obs*_ in eq. (9), and the rest of the total Hamiltonian (the interaction between atoms and cavities, and the interaction between cavities and fibers) as 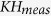 in eq. (9). Then, choosing the “dark Zeno subspace”, that means 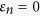 in eq. (10), we can obtain an effective Hamiltonian named the Zeno Hamiltonian 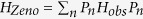. (2) For this effective Hamiltonian, by using TQD, we construct the CDD Hamiltonian *H*(*t*) that speeds up the adiabatic process. (3) Similar to the GHZ state generation, we find out the corresponding non-resonant system (the APF Hamiltonian) whose effective Hamiltonian 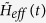 has the form in eq. (22). (4) Making 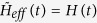, the parameters for the APF Hamiltonian are determined and the shortcut is constructed. Then the APF Hamiltonian would govern the system to achieve the same final result as the adiabatic process governed by the original Hamiltonian with a much shorter operation time. Similar idea can be generalized to generate other multi-qubit quantum entangled states, for example, Bell states, *W* states, singlet states, and so on. This might lead to a useful step toward realizing fast and noise-resistant quantum information processing for multi-qubit systems in current technology.

## Additional Information

**How to cite this article**: Chen, Y.-H. *et al*. Shortcuts to adiabatic passage for fast generation of Greenberger-Horne-Zeilinger states by transitionless quantum driving. *Sci. Rep.* **5**, 15616; doi: 10.1038/srep15616 (2015).
